# School Health Education Program in Pakistan (SHEPP): findings from a feasibility trial in pre-adolescent school children from a lower middle-income country

**DOI:** 10.1186/s40814-023-01344-9

**Published:** 2023-07-17

**Authors:** Aysha Almas, Romaina Iqbal, Abdul Ghani, Zainab Samad, Sania Sabir, Khawar Kazmi

**Affiliations:** 1 Department of Medicine, Aga Khan University, Karachi, Pakistan; 2 Department of Community Health Sciences, Aga Khan University, Karachi, Pakistan; 3 Institute of Public Health, Quetta, Pakistan; 4 National Institute of Cardiovascular Diseases, Karachi, Pakistan

**Keywords:** Diet, Health education adolescents, Cardiovascular, School, Feasibility, Trial

## Abstract

**Background:**

The school environment plays an essential role in promoting health education and physical activity for children and adolescents. We aim to assess the feasibility of threefold health education program in children and its potential efficacy on physical activity and diet and cardiometabolic risk factors including blood pressure, body mass index (BMI), and waist circumference.

**Methods:**

The SHEPP was a parallel group feasibility intervention trial conducted in two schools over 23 months. All children aged 9–11 years enrolled in the schools were included. The SHEPP intervention comprised of health education on healthy lifestyle and physical activity sessions for children, training of teachers, and awareness sessions for parents conducted over 10 months. One school received the intervention of SHEPP while the other school continued routine activity. The primary outcome was the feasibility of SHEPP in terms of recruitment, retention, and treatment fidelity. Secondary outcomes were physical activity levels, dietary intake (of fruits and vegetables), and cardiometabolic risk factors (blood pressure, BMI, and waist circumference (WC)).

**Results:**

A total of 1280 preadolescent children were assessed for eligibility and 1191 were found eligible. The overall recruitment *n* (%) was 982/1191(82.5%) with 505(51.4) from SHEPP intervention school and 477(48.6) in routine activity school. The overall retention rate* n* (%) at 10-month follow-up was 912/982(92.8), with 465/505(92) in SHEPP intervention school and 447/477(93.7) in routine activity school. In treatment fidelity, 132/144(92) %). Physical activity sessions and all (100%) health education sessions were conducted for each of the twelve classes. Mean (SD) Seven-day Physical activity increased by 134 (196) min in the SHEPP intervention school v 29.8(177) in the routine activity school (*P* value < 0.001) from baseline to follow-up. Overall, there was an increase in vegetable intake (> 3 serving /day) in SHEPP intervention school of 5.5 to 21.4% from baseline to follow-up compared to 7.5 to 14.9% in routine activity school. The mean change (SD) in systolic blood pressure was 1.3(12) mmHg, 2.2(19.0) mm Hg in in diastolic blood pressure, − 0.09(5.4) kg/m^2^ in BMI and 6.2 cm in waist circumference in the intervention arm versus − 3.4(11.1) mm Hg in SBP, − 4.3(9.9) mm Hg in DBP, − 0.04((4.6) kg/m^2^ in BMI, and 3.8 cm in WC in the control arm.

**Conclusion:**

We found that intervention using SHEPP is feasible in schools and may help children to adopt a healthy lifestyle as they age by increasing physical activity. However, the potentially beneficial effect on diet, MI, and BP needs further exploration and a longer follow-up, more specifically at the juncture of teenage and adulthood.

**Trial registration:**

NCT03303287.

## Key messages regarding the feasibility

- Feasibility of a school health education program in preadolescents while including parents and teachers is not known in a lower-middle-income country like Pakistan.
- This study shows that theschool health education program in Pakistan is feasible with good recruitment, acceptability, and retention
- These findings imply that a larger hybrid implementation trial in with better engaging strategies for parents and more extensive healthy lifestyle training for teachers.

## Background

Globally, adolescents demonstrate multiple modifiable risk factors that can lead to the development of non-communicable diseases (NCDs). Eighty-two percent of adolescents are at risk of developing NCDs due to physical inactivity and low fruit and vegetable intake [1]. Hence, NCDs have emerged as a global priority in the Sustainable Development Goals [2]. Health literacy is considered by the World Health Organization (WHO) to be one of the pillars of health promotion and a critical determinant of health for people’s empowerment [3]. Health education and literacy in children and adolescents is even more challenging for lower middle-income country like Pakistan where the health literacy is low [4] and a formal school health education system is non-existent.

High childhood and adolescent body mass index (BMI) is associated with increased risk of cardiovascular disease in adulthood [5]. Thirteen percent children from Karachi, Pakistan have been observed to be obese (higher BMI) and 21% had greater abdominal obesity [6]. In Pakistan, only 7% of the girls and 30% of the boys aged 13–14 years do the recommended physical activity of 1 h per day. About 66% of the children attending school reported that they did not participate in organized sports within school and 85% had a largely a sedentary lifestyle [7–9]. Increased interest in watching television or playing on computer, lack of safe outdoor playgrounds and walking tracks in major cities, residing in urban areas are some of the other important factors responsible for suboptimal physical activity levels [10–12]. Consumption of discretionary food items and sugar sweetened beverages were more than fruits and vegetable intake in adolescent school children from Karachi, Pakistan [13, 14]. Prevalence of smoking was reported to be 14% in pre-adolescents and adolescents aged 5–15 years from Pakistan [15, 16]. Hence risk factors for NCDs is quite prevalent in children and adolescents in Pakistan, thus mandating a robust, innovative, scalable school health education program. While School health education program has been endorsed and emphasized by the WHO, it has not been implemented in true spirit in Pakistan. This is reflected by the fact that specific health education module or 30 min physical activity sessions at least 3–4 days per week is not practiced in schools.

Theory of planned behavior is one behavior change theory that has demonstrated usefulness in studies of health-related behavior [17]. A review on the contextual influences on physical activity and eating habits of the community level reports that successful community-based health promotion strategies should consist of multilevel—multicomponent interventions on different level of environments [18–20]. Based on this, we planned this three-fold intervention including children, parents, and teachers as children learn behaviors from both school and home environment. Also, using multiple strategies like health education interactive session and physical activity session simultaneously might help in changing behaviors. We have previously shown the feasibility of a school-based physical activity program in a public sector girl’s school of urban Pakistan showing a favorable trend on BP and BMI at follow-up [21]. However, the study did not include any health education intervention targeting children, teachers, or parents. We here report the feasibility of a three-fold, School Health Education Program in Pakistan (SHEPP) and potential efficacy on physical activity, diet, and cardio metabolic risk factors including BP, BMI, and waist circumference.

## Methods

**Fig. 1:**
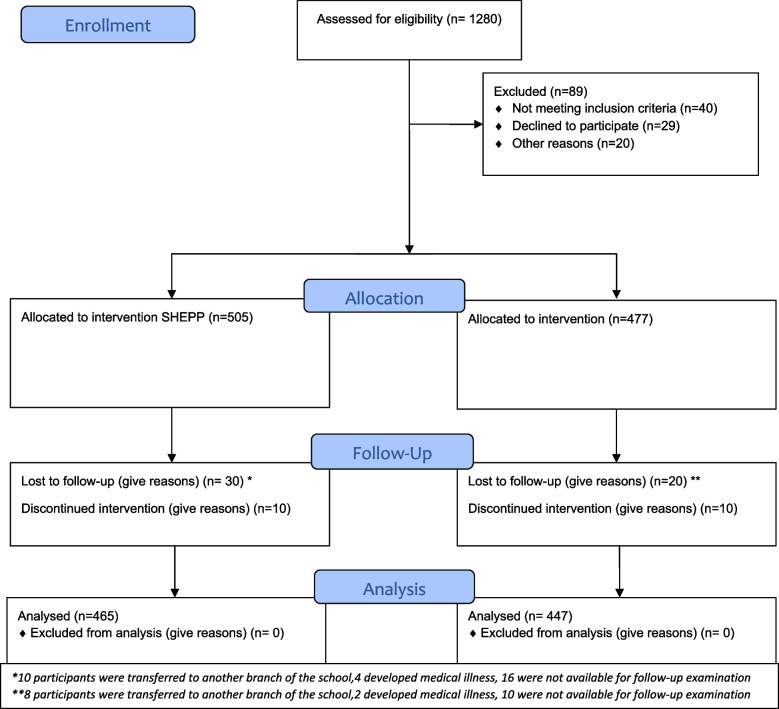
Study flow at baseline and follow-up at 12 months in the school health education program of Pakistan (SHEPP). *10 participants were transferred to another branch of the school,4 developed medical illness, 16 were not available for follow-up examination **8 participants were transferred to another branch of the school,2 developed medical illness, 10 were not available for follow-up examination

The SHEPP was a parallel group feasibility intervention trial conducted from September 2017 to November 2019 including 10 months SHEPP intervention. The details of methods and interventions have been published earlier [22]. In brief, it was conducted in two schools located in lower- to middle-income class, at different locations in Karachi but following similar school curriculum under the Aga Khan Education Service, Pakistan. These two schools were selected based on convenience on being close to the study center and link to Aga Khan Education service. One school was allocated to SHEPP intervention while the other school was assigned to carry on routine activity. All children aged 9–11 years enrolled in the above-mentioned schools were included. The rationale for this age group is that in this period of 9–11 years age, physical activity starts to decline mainly in girls [23]. The study flow is shown in Fig. 1. Those suffering from any physical disability were excluded from the study however they attended health education teaching sessions. Also, efforts were made to include them in any physical activity which they could perform depending on their disability. Out of the 10 classes in each school, children fulfilling the eligibility criteria i.e., 9–11 years (class 2, 3, and 4) were approached through school administration. Within each class in the respective school there were 40 children. Ethical approval from the ethics review committee, Aga Khan University was obtained (ERC number 2571-Med-ERC-13, 2019–0721-3963). Informed consent and assent were taken from parents and children respectively by the resaerch staff.

### SHEPP intervention

The SHEPP was designed focusing on children mainly while involving teachers and parents at the same time in order to overcome cultural, socioeconomic limitations in performing physical activity [24]. The outline of the SHEPP intervention and the content was designed by a team comprising of principals of schools, teachers, physical trainer of the school, physician health experts including cardiologist, nutrition epidemiologist, and research specialist. At least 6 separates 1-h meetings were conducted with this team individually or in groups by the PI and the study team over a period of 4–5 months. After that, a draft SHEPP was made which was shared with the team for feedback, before initiating the study.

It was conducted over a 10-month period (August 2018 to May 2019) within school premises in one school while routine activities were carried out in the control school. The 10 months cutoff was chosen as benefits on cardiovascular risk factors are more pronounced at 10-month follow-up [25].

The *SHEPP-Children* comprised of physical activity sessions and healthy heart teaching sessions.

The SHEPP physical activity sessions were conducted by 2 research staff trained in delivering physical activity sessions by the PI and an expert physical trainer over 2 weeks. The assigned school physical trainer shadowed with this research staff for first 3 classes and then independently delivered physical activity intervention. Data monitoring was done by random checks on the field site by PI while data was being collected by the research staff. Additionally, a logbook was maintained to log all the healthy heart educational sessions and physical activity sessions. To improve adherence physical activity leaders were selected from each class and were given a badge of physical activity champion. Additionally, after completion of initial 12 sessions of physical activity, children were asked “If they had any other games they wanted to play” and these were incorporated into the SHEPP Physical activity sessions The SHEPP-Physical Activity sessions consisted of 12 different activities of 10–15 min structured exercise and 15–20 min games,10 min physical activity daily during assembly *and* 1 min between class physical activities*.* Details of physical activity sessions have been published earlier [22].

*The SHEPP healthy heart teaching* was done by the 1 research staff who was trained by the PI for 2 weeks. The teachers shadowed the research staff during these sessions but did not deliver the intervention independently. In addition to the SHEPP for teachers, these sessions provided hands-on training to teachers for conducting these sessions in the future independently. *SHEPP Healthy Heart teaching for children* comprised of 6 sessions of 30 min each which took place within school time, including: (i) a happy heart; what makes heart happy and what makes it sad; (ii) the smart diet; (iii) keep moving; (iv) smoking cigarette or chewing “gutka “is bad for health; (v) stay clean, stay healthy; (vi) pax good behavior game. These were interactive sessions using brief pictorial presentations, videos, and an activity to make it interesting for the children. Stay clean and PAX good behavior game (PAX GBG) was included to make it a more holistic learning experience for the children based on a “need” for the school children in preliminary discussions with teachers [26, 27]. The PAX GBG divides the class into teams and those which demonstrate bad or spleen behaviors are given marks. The team which gets less marks i.e., demonstrates good behavior wins and is rewarded through a 1-min exercise. Examples of good behaviors are raising hand when asking teacher or doing work quietly while example of spleem behavior is making noise or speaking without permission of teacher [28]. Health education posters focused on healthy heart were displayed in each class also.

To integrate both the SHEPP physical activity and healthy heart sessions for children in the timetable of each class, they were reorganized in such a way that the healthy heart sessions were taken in the designated extracurricular activity periods of the class. This was important so that the regular teaching and course work is not affected. The SHEPP PA sessions took place in the designated physical activity periods. This arrangement and reorganization of timetable required 1 h period of teachers every month in collaboration with the study team.

The *SHEPP for Teachers* was a 3-h interactive workshop conducted on weekends, based on the same core topics as SHEPP Healthy Heart teaching for children; however, they were slightly modified according to adult learning need of teachers as follows: (i) The Heart attack!; (ii) What is Healthy diet; (iii) Keep moving and stay active; (iv) Why smoking, “gutka, shisha and chaalia” is bad; (v) Wash your hands always!; (vi) Stay calm, stay away from anger and stress. The teaching methods included (1) presentations by facilitators; (2) interactive discussion between facilitators and teachers; (3) 3-min healthy physical activity during workshop; and (4) discussion on personal stories of teachers. These sessions were taken by the PI and a research specialist and were taken on weekends in which teachers came to school to complete other academic assignments. The purpose of these sessions is so that the teachers will have good knowledge base, they can facilitate physical activity and healthy eating behavior in children and themselves.

The *SHEPP Parents* was a 3-h Healthy heart awareness session on the same core topics as for SHEPP for Teachers; however, it was a lecture-based session with questions in the end. The objective of these sessions for parents was to have the basic awareness about healthy lifestyle, so they can facilitate the same in their children. These sessions were taken by the PI and the research specialist. Parents were invited twice on a weekend to attend, and some sessions were tagged with the parent teacher meeting. To encourage participation in the workshop a free health checkup for the teachers including blood pressure measurement, glucose monitoring, height, and weight measurements (BMI calculation) were also be offered to both parents and teachers. These services were provided by other allied health workers including health care assistants and pharmacists, not part of the study team.

### SHEPP-routine activity school

The routine activity (control group) carried on routine physical activity in school which is 30 min physical activity as per schedule in school. For ethical reasons (1) a workshop for teachers was held as for SHEPP teachers in the intervention arm towards the end of the study. (2) Health Education posters were placed in school classes towards the end of the study. (3) A large class format of combined health tips (on physical activity and diet) was held for all children after completion of study.

### Assignment of interventions

This was a non-randomized study. One school was allocated to SHEPP intervention after baseline data collection while the other school carried on routine activity and were subjected to health education after follow-up data collection. The decision to select an intervention school is based on convenience and not randomized. The data collectors who collected data were blinded to the assignment of intervention. The physical activity trainer conducted sessions in the intervention school and had no role during the data collection part of the study.

### Outcome measures

#### Measures of feasibility

The feasibility of SHEPP was assessed in terms of recruitment, retention, and treatment fidelity. *Recruitment* was defined as the percentage of participants enrolled out of the total participants who were invited at baseline. *Retention* is defined as the percentage of participants who were available for follow-up at 10 months out of those recruited at baseline. *Treatment fidelity* is a measure of the reliability of the administration of an intervention in a treatment study. It is an important aspect of the validity of a research study [21, 29]. *Treatment fidelity* was defined as proportion of physical activity sessions conducted out of the total planned [21]. The trial was considered as feasible if recruitment, retention and treatment fidelity are > 70% [30].

#### The measure of potential effect

Physical activity levels (in school, out of school, moderate to vigorous physical activity and sedentary time) was assessed by change in time (minutes) spent in physical activity from baseline to follow-up. Dietary patterns in terms of percentage increase in fruits and vegetable serving, and percentage decrease in sweetened beverages and snacks/day and change cardiometabolic risk factors (change in blood pressure, BMI, and waist circumference) were recorded.

#### Sample size

Since this is a parallel group feasibility intervention trial in school setting, where interventions can only be done at class level (in a group) and not individually due to ethical and administrative reasons, we included 12 sections of classe 2, 3 and 4 of children from the intervention school and similar 12 sections from control school. Each class has approximately 40–45 students making a total sample size of approximate 540 participants in intervention and 540 in control arm. This sample size is sufficient to measure Feasibility outcomes of 70% recruitment, retention, and treatment fidelity. As mentioned by Thabane et al., for an expected completion rate (recruitment, retention and treatment fidelity for this study) of 75%, the minimum required sample for the pilot study would be at least 75 participants using a 95% CI for the proportion and a margin of error of 0.05, a lower bound of this CI of 0.70 [31]. Hence, the above mentioned sample size for this study is sufficient to assess the feasibility outcomes.

### Data collection

Data collection was done by 2 research staff who were trained by the primary Investigator over 2 weeks for (a) filling data collection forms (b) measurement of blood pressure, waist circumference, height, and weight(d)collecting data for 24-h dietary recall. Assessment of outcomes was done at baseline and then after 10 months of intervention by these trained research staff who were blinded to allocation of intervention to the schools. Physical activity was assessed using the validated youth Physical Activity Questionnaire modified for children at baseline and follow-up by trained research staff [32]. Details of the modified version of YPAQ used in SHEPP has been reported earlier [22]. Physical activity was also measured objectively using Mi wrist bands for at least 5% (*n* = 60) of the participants, to check the validity of physical activity measured by the questionnaire [33]. However, the results will be reported separately. Dietary assessment was done using a 24 h dietary recall on a typical weekday done by trained research staff while asking questions from preadolescent children. Number of raw fruit and vegetables, number of sugar sweetened beverages and snacks/day were recorded at baseline and follow-up through these dietary recalls. Standard measures were used to record blood pressure by using Omron m5 monitors using with a pediatric cuff by the trained research staff [34]. Weight was recorded using Tanita’s digital weight scales. Community-setting aluminum scale were used to measure height with subjects standing barefooted (without head cover). The data management has been reported earlier in detail [22]. Measurement of blood pressure, height, weight, and waist circumference was done twice for each child and both readings were recorded to address possible reporting bias.

#### Adverse events

During the exercise any injury, vasovagal episodes were noted down by the physical trainer and were informed to the PI and the participant were provided first aid in the school clinic. If participants were unable to attend three sessions consecutively then they were considered non-adherent. Any participant who discontinues participating in the intervention will be considered as drop out.

#### Data analysis

Mean (SD) was used to report quantitative variable and frequency and percentage for categorical variable. Recruitment, retention, and treatment fidelity is reported as percentage. Student’s *t* test was used to compare quantitative variables (change in physical activity, BP, BMI, and waist circumference before and after intervention) between groups. Chi-square test was used to compare qualitative variables where applicable between the two groups from baseline to follow-up. Analysis was done on intention to treat basis that is all participants will be analyzed in the same group as at time of allocation.

## Results

### Feasibility of SHEPP

#### Recruitment

A total of 1280 children were assessed for eligibility and the consent forms and assent forms were distributed to 1191 eligible parents and their children. The recruitment n (%) was 982/1191(82.5). The recruitment from SHEPP intervention school was 505(51.4) and 477(48.6) from routine activity school. Mean (SD) age of the recruited children was 9.6(0.9) years. Among those recruited 496(50.5) were boys and 486(49.5) were girls. Baseline characteristic of the children is shown in Table 1.

**Table 1:** Characteristics of adolescent school children at baseline

	Overall	Intervention school	Control school	*P* value
	*N* = 982	*n* = 505(51.4)	*n* = 477(48.6)	
Mean age (SD)	9.6(0.9)	9.55(0.8)	9.7(1.0)	< 0.001
Age categories				
8–9 years old	395(40.2)	254(50.5)	141(29.6)	
9–10 years old	534(54.4)	243(48.3)	291(61.1)	
10–11 years old	50(5.1)	6(1.2)	44(9.2)	< 0.001
Gender				
Boys	496(50.5)	258(51.1)	238(49.9)	
Girls	486(49.5)	247(48.9)	239(50.1)	0.7
Class				
Class II	416(42.4)	257(50.9)	159(33.3)	
Class III	415(42.3)	248(49.1)	167(35)	
Class IV	151(15.4)	-	151(32)	-
Parents employment status^a^				
Only father	733(74.6)	390(77)	343(72.1)	
Both mother and father	226(23)	100(19.9)	126(26.5)	
Only mother	16(1.6)	11(2.2)	5(1.1)	
None of the parent	3(0.3)	1(0.2)	2(0.4)	-

^a^4 children could not respond to the job status of their parents

#### Retention

The overall retention rate n (%) at 10-month follow-up was 912/982(92.8), with 465/505(92) in SHEPP intervention school and 447/477(93.7) in routine activity school. Figure 1 shows the consort flow diagram of the study flow.

#### Treatment fidelity

Children

(i) Twelve physical activity sessions of 30 min were planned for each of the 12 sections of classes 2, 3, and 4 and 132/144(92) %)) of them were conducted. Twelve sessions were missed on the designated time due to exams of children or leave but were conducted later in extra physical activity class. (ii) The 10 min physical activity session in the assembly could not be done due to changes in school policies during the intervention. (iii) One minute between class physical activities were implemented in the initial 8 weeks of the intervention and later the designated physical activity champions were asked to lead these between class activities. (iv) Similar 6 health education session for the 132 classes were all (100%) conducted.

Teachers

Two, 3-h workshops were done for all schoolteachers in both SHEPP intervention school and routine activity school (after collection of follow-up data). The attendance in these sessions were 30/35(86%) in SHEPP school and 25/30(83%) in routine activity group.

Parents were invited twice on a weekend to attend a 3-h healthy heart teaching in the SHEPP intervention school, but the attendance was very low; only 64/250(25) parents participated in the healthy heart teaching sessions.

### Measure of potential effect on Physical activity, diet, and cardio metabolic outcomes

#### Seven-day physical activity

Overall, the mean (SD) change in 7-day physical activity in minutes from baseline to follow-up was as follows: there was increase by134(196) min in the SHEPP intervention school v 29.8(177) in the routine activity school (*P* value < 0.001).The increase in out of school physical activity component of physical activity was more compared to in school physical activity from baseline to follow-up (101.0(187) v 33(27)). Also, the change(increase) in time spent in sedentary activity from baseline to follow-up was less in SHEPP intervention school compared to routine activity school ( 200(583) v 482(628)), *p* value < 0.001). Table 2 shows 7-day physical activity at baseline and 10-month follow-up in adolescent school children. On comparison of 7-day physical activity at 10 months follow-up there was no difference in physical activity of boys and girls in SHEPP intervention school {(429(150) v 433(171), *p* value 0.7} and routine activity school {381(138) v 398(162), *p* value 0.2}. The Cronbach alpha of YPAQ ranged from 0.50 to 0.52 indicating moderate level of reliability.

**Table 2:** Seven-day physical activity at baseline and 10-month follow-up in adolescent school children

	Baseline	Follow-up *N* = 982)	Change^a^	*P* value^b^
	Mean (SD)	Mean (SD)	Mean (SD)	
Total physical activity in minutes				
Intervention	297.6(143)	430.9(161)	134(196)	
Control	362.3(131)	389.4(151)	29.8(177)	< 0.001
In school physical activity in minutes				
Intervention	45.1(24)	77.8(17)	33(27)	
Control	50.1(22)	90.5(22.5)	40.2(28.1)	< 0.001
Out of school physical activity in minutes				
Intervention	252.4(133)	353(156)	101.0(187)	
Control	312.1(126)	298(145)	− 10.3(172)	< 0.001
Moderate to vigorous physical activity in minutes				
Intervention	88.5(62.5)	130.6(72.5)	46.6(97.8)	
Control	104.9(67)	127(73.6)	28(86)	0.07
Sedentary activity in minutes				
Intervention	6292(372)	6494(501)	200(583)	
Control	6032(457)	6509(501)	482(628)	< 0.001

^a^Change = follow-up minus baseline physical activity

^b^*P* value between change PA between intervention and control

#### Dietary intake of various food groups in 24 h

**Fig. 2:**
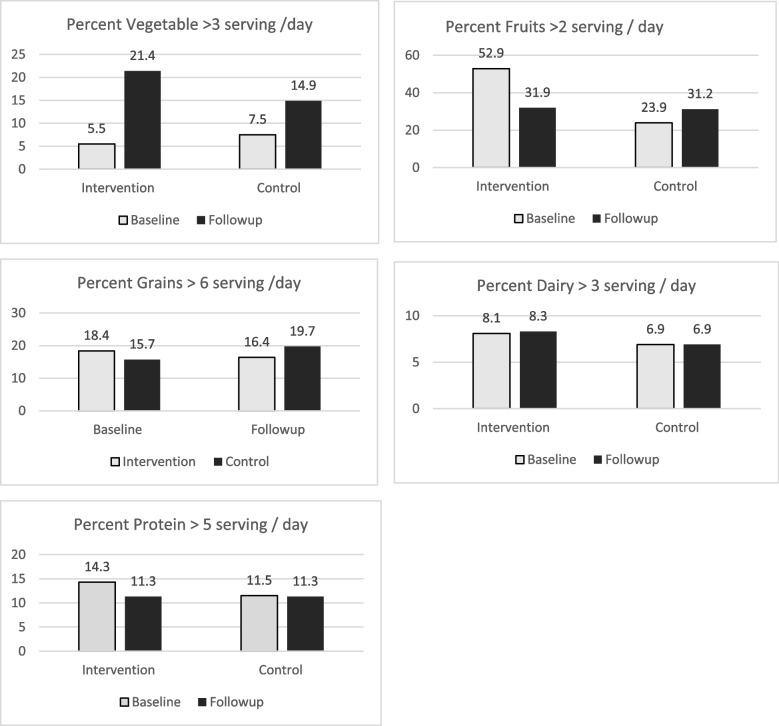
Dietary intake of various food groups in 24 h at baseline and 10-month follow-up in adolescent school children

Overall, there was increase in vegetable intake *n* (%) (> 3 serving/day) in SHEPP intervention school (28(5.5) to 108(21.4)) from baseline to follow-up compared to routine activity school; 36(7.5) to 71(14.9). Figure 2 shows dietary intake of various food groups in 24 h at baseline and 10-month follow-up in adolescent school children. There was increase in number of sugar sweetened beverage consumed in both SHEPP Intervention school (1 to 1.4) and routine activity (1 to 1.6) from baseline to follow-up. There was reduction in mean (SD) consumption of readymade food items in both SHEPP intervention School 2(2.2) to 1.6(1.8) and routine activity 2(2.0) to 1.6(1.7) from baseline to follow-up.

#### Cardio metabolic outcomes

The SHEPP did not show reduction in blood pressure or waist circumference in the intervention school compared to routine activity school at 10 months follow-up. There was reduction in BMI of − 0.09(5.4) kg/m^2^ in intervention and -0.04((4.6) kg/m^2^ in the control school from baseline to follow-up (*P* value 0.8) in the SHEPP intervention school compared to routine activity school (Table 3). No adverse events including injury or vasovagal episodes were reported during SHEPP intervention.

**Table 3:** Cardio metabolic outcomes at baseline and 10-month follow-up in adolescent school children

	Baseline	Follow-up *N* = 982)	Change^a^	*P* value^b^
	Mean (SD)	Mean (SD)	Mean (SD)	
Systolic blood pressure (mm Hg)				
Intervention	101(10.6)	102.3(11.2)	1.3(12)	
Control	107(10.8)	104(9.8)	− 3.4(11.1)	< 0.001
Diastolic blood pressure (mm Hg)				
Intervention	67.3(9.3)	70(17.7)	2.2(19.0)	
Control	73(8.6)	68.6(8.7)	− 4.3(9.9)	< 0.001
Body mass index kg/m^b^				
Intervention	16.2(3.1)	16.1(6.4)	− 0.09(5.4)	
Control	17.0(3.1)	16.9(5.8)	− 0.04((4.6)	0.8
Waist circumference (cm)				
Intervention	61.4(7.7)	68(9.5)	6.2	
Control	65(8.3)	69(9.1)	3.8	< 0.001

^a^Change = follow-up minus baseline cardiovascular outcomes

^b^*P* value between change in cardiovascular outcomes between intervention and control

## Discussion

We report the feasibility of SHEPP, based on three-fold health education program in preadolescent school children in Karachi. Our findings highlight that SHEPP is feasible in terms of recruitment (82%), retention (92%), and treatment fidelity (92%). However, only 25% of parents participated in health awareness sessions highlighting a major gap in this threefold intervention. The SHEPP was able to increase 7 days total physical activity time by 134 min, out of school physical activity time by 100 min, reduction in sedentary time by 200 min and increase in MVPA by 46 min, in the SHEPP intervention school versus routine activity school. The increase in proportion of preadolescent children 24-h vegetable intake was more in SHEPP intervention compared to routine activity. The SHEPP could not demonstrate and effect on cardio metabolic risk factors including BP, BMI, and waist circumference.

A cluster randomized feasibility trial in eight primary schools (*n* = 358 pupils)from the North of England reports a retention rate of 93% at 6 months follow-up [35]. In another feasibility cluster, non-RCT designed study (*n* = 64) in 2 schools from China the recruitment and retention rates at 16 weeks were 100% [36].We have previously shown from a two-arm parallel cluster intervention trial (*n* = 280), recruitment of 82% and retention of 79%. Our current recruitment from the SHEPP study is 82% and retention at 10 months follow-up is 93%. Hence showing that this study was feasible in terms of recruitment and retention rates in comparison to previous study. Our recruitment and retention rates are better than our previous study reported in 2013 [21]. The reason for this improvement in feasibility in SHEPP could be (i) the use of a threefold intervention focused on children, parents and teachers. (ii) Addition of a health education component (+ physical activity) as opposed to an only physical activity component. This emphasizes the importance of a holistic health education strategy encompassing healthy lifestyle while addressing both the school and home environment. This is in line with the theory of planned behaviors using multilevel-multicomponent interventions on different levels of environments including children, parents, and teachers [18, 20]. These concepts have further been endorsed in a systematic review and meta-analysis on 10,871 children and adolescents from 80 studies [37, 38]. These studies support multicomponent interventions and also suggested that a comprehensive school health program including changes in physical exercise curricula and school nutrition policies to prevent childhood obesity is necessary.

In the Camp Nutrition Education Recreation and Fitness study from 9 schools and 81 children from OH, USA, the treatment fidelities delivery of the intended lesson ranged between 79 and 90% [39]. In the Healthy Lifestyles Program (HeLP) study, a novel, behavior change school-located intervention for 9–10 years old, conducted in 16 schools and 676 children, the treatment fidelity ranged from 94 to 100%. Additionally, the study also measured quality of delivery score of these sessions, which was scored 9/10 by the children [40]. The treatment fidelity in the SHEPP study was 92% which is comparable to the figures reported in the former 2 studies. While we did achieve 92% treatment fidelity for conducting health and physical activity sessions, we could not conduct 10 min physical activity daily during assembly due to limitations in the school’s logistics. Additionally, these fidelity results are limited by the fact that we could not measure fidelity beyond intervention delivery [41]. Future studies need to use quality measures for the intervention delivered using quantitative scores or qualitative surveys for better understanding in our setup.

Participation of parents in health education sessions targeted towards their 8–10 years old children (and themselves) has been suboptimal [42]. In a randomized controlled trial focused on obese children and their parents(*n* = 249), testing Behavioral family-based versus parents only versus health education condition, showed that there was significant decrease in BMI in the parents only group. However, regular attendance of parents and families was a major challenge in the maintenance phase of the study. Our study showed that the attendance in the health awareness sessions was only 25%, which reflects the lack of commitment by parents towards gaining better understanding towards healthy lifestyle of their children. The reasons for this could be conflicting schedules with work, although only 23% of children had both parents working in our study. Secondly, parents might perceive that giving healthy lifestyle to children is government responsibility in some instances [43]. Thirdly parents from lower socio-economic position might find it challenging to provide healthy diet including fruits and healthy breakfast to their children [44].

In the Camp Nutrition Education Recreation and Fitness study, overall proportions of participants achieving sufficient moderate (5,·24%) and sufficient vigorous (4,·36%) physical activity improved, an overall decrease (12,·99%) in the proportion of participants who engaged in insufficient physical activity from baseline to post-intervention [39]. However, the study could not show any effect on vegetable, fruit, or snack preference outcomes. In the feasibility cluster non-RCT designed study (*n* = 64) in 2 schools from China, there was no increase in the 7-day steps in the intervention group compared with the control [36]. In a school- and home-based intervention to improve adolescents’ physical activity and healthy eating from USA (*n* = 81 parent–adolescent dyads), the three component intervention, autonomous motivation for physical activity and self-efficacy for healthy eating were significantly higher in the intervention than control group [45]. While SHEPP was a feasibility study the intervention was able to show increase in out of school physical activity time, reduction in sedentary time and increase in MVPA and increase in vegetable intake in the intervention school.

The strength of the study is that it was a feasibility trial which was conducted on a relatively large sample (*n* = 1191) of adolescents from 2 private schools in lower- to middle-income neighborhoods in Karachi. The trial also measured the potential effect on healthy behaviors including physical activity and diet. However, the trial has several limitations. Firstly, self-reported physical activity was used as opposed to using objective measures like accelerometers or wrist bands. Since accelerometers are expensive and cumbersome to wear by adolescents, we did measure physical activity using wrist bands on 5% of the sample [46]. Also, Cronbach alpha 0.50–0.52 for the modified youth Physical Activity Questionnaire is low which is a limitation. Although the same tool was used at baseline and follow-up, and it showed that physical activity improved in intervention arm. Secondly, the findings from this study cannot be generalized to government sector schools as they have even more limited resources of conducting healthy lifestyle interventions. However, SHEPP will help in outlining a similar program for these schools on a larger scale. Thirdly, the parental involvement component was weak as it was able to engage only one fourth of the parents. Better coordination with parents, using social media and devising robust, easily accessible health education and screening programs tied with adolescent education needs to be explored [47]. We also could not demonstrate any potential effect on health outcome including blood pressure and weight. However, studies on effect of exercise on reduction in resting blood pressure show mixed results [48, 49]. The reason for this could also be that the age of 9–11 years is also the age of puberty which might have direct effect on increasing blood pressure [50]. Also, specific physical activity for disabled children by using additional aids might be useful in future studies. There was no formal competency assessment of the teachers on their understanding of the SHEPP due to resource and time constraints, but ideally this should be incorporated in future larger studies. This is a feasibility study, but we did not use mixed method design to assess this. Future studies need to incorporate qualitative methods to understand the perceptions, barriers, and facilitators in implementing such a program. The number of 11-year-olds or those in class IV preadolescents were lower in both groups, more in the intervention arm and this could have resulted in selection bias. However, the 9- and 10 years old preadolescents were same in both intervention and control arm but finding might need further exploration in a larger sample of 11-year-olds. The reason for this was that both schools differed in the number of sections for each class.

## Conclusions

We found that intervention using SHEPP is feasible in schools and may help children to adopt a healthy lifestyle as they age by increasing physical activity. However, the potentially beneficial effect on BMI and BP possibly needs a longer follow-up, more specifically at juncture of teenage and adulthood. The implications of these findings are that a larger hybrid design implementation trial be planned with better engaging strategies for parents and a more extensive healthy lifestyle training for teachers.

## Data Availability

The datasets used and/or analyzed during the current study are available from the corresponding author on reasonable request.

## Abbreviations

PA: Physical activity
WC: Waist circumference
NCD: Non-communicable disease
SHEPP: School Health Education Program in Pakistan
